# Identification of a novel major locus for gray leaf spot resistance in Italian ryegrass (*Lolium multiflorum* Lam.)

**DOI:** 10.1186/s12870-014-0303-6

**Published:** 2014-11-18

**Authors:** Wataru Takahashi, Yuichi Miura, Tohru Sasaki, Tadashi Takamizo

**Affiliations:** Forage Crop Research Division, NARO Institute of Livestock and Grassland Science, 768 Senbonmatsu, Nasushiobara, Tochigi 329-2793 Japan; Kyushu Experiment Station, Japan Grassland Agriculture and Forage Seed Association, 1740 Takaba, Koshi, Kumamoto 861-1114 Japan; Forage Crop Research Institute, Japan Grassland Agriculture and Forage Seed Association, 388-5 Higashiakada, Nasushiobara, Tochigi 329-2742 Japan; Present address: Snow Brand Seed Co., Ltd, Hokkaido Research Station, 1066 Horonai, Naganuma-cho, Yubari-gun, Hokkaido 069-1464 Japan; Present address: Hokkaido Branch, Japan Grassland Agriculture and Forage Seed Association, 406 Higashi-Nopporo, Ebetsu, Hokkaido 069-0822 Japan

**Keywords:** Blast, Comparative genomics, Expressed sequence tag, *Lolium multiflorum*, *Magnaporthe oryzae*, Single-strand conformation polymorphism

## Abstract

**Background:**

Gray leaf spot (GLS), caused by *Magnaporthe oryzae* (anamorph *Pyricularia oryzae*), in ryegrasses is a very serious problem. Heavily infected small seedlings die within a matter of days, and stands of the grasses are seriously damaged by the disease. Thus, the development of GLS-resistant cultivars has become a concern in ryegrass breeding.

**Results:**

Phenotypic segregations in a single cross-derived F_1_ population of Italian ryegrass (*Lolium multiflorum* Lam.) indicated that the GLS resistance in the population was possibly controlled by one or two dominant genes with 66.5–77.9% of broad-sense heritability. In bulked segregant analyses, two simple sequence repeat (SSR) markers, which have so far been reported to locate on linkage group (LG) 3 of Italian ryegrass, showed specific signals in the resistant parent and resistant bulk, indicating that the resistance gene locus was possibly in the LG 3. We thus constructed a genetic linkage map of the LG 3 covering 133.6 centimorgan with other SSR markers of the LG 3 of Italian ryegrass and grass anchor probes that have previously been assigned to LG 3 of ryegrasses, and with rice expressed sequence tag (EST)-derived markers selected from a rice EST map of chromosome (Chr) 1 since LG 3 of ryegrasses are syntenic to rice Chr 1. Quantitative trait locus (QTL) analysis with the genetic linkage map and phenotypic data of the F_1_ population detected a major locus for GLS resistance. Proportions of phenotypic variance explained by the QTL at the highest logarithm of odds scores were 61.0–69.5%.

**Conclusions:**

A resistance locus was confirmed as novel for GLS resistance, because its genetic position was different from other known loci for GLS resistance. Broad-sense heritability and the proportion of phenotypic variance explained by the QTL were similar, suggesting that most of the genetic factors for the resistance phenotype against GLS in the F_1_ population can be explained by a function of the single resistance locus. We designated the putative gene for the novel resistance locus as *LmPi2. LmPi2* will be useful for future development of GLS-resistant cultivars in combination with other resistance genes.

**Electronic supplementary material:**

The online version of this article (doi:10.1186/s12870-014-0303-6) contains supplementary material, which is available to authorized users.

## Background

Italian ryegrass (*Lolium multiflorum* Lam.) originated in the Mediterranean region and is produced mainly for hay and silage. It is one of the most important forage grasses in the temperate zones of Europe and Asia because of its high palatability to and digestibility by livestock [1,2].

Blast disease, caused by the fungal pathogen *Magnaporthe oryzae* (anamorph *Pyricularia oryzae*), is the most severe disease of rice. Blast may cause devastating production losses in rice in epidemic years. Thus, many researchers have studied rice blast disease using genetic, pathological, and biotechnological approaches for controlling outbreaks of the disease by determining many aspects of the resistance mechanisms in rice and the pathogenicity of the disease [3].

Ryegrass blast, also called gray leaf spot (GLS), has recently become a very serious problem in Italian ryegrass in Japan [4] and in perennial ryegrass (*L. perenne* L.) in the United States [5]. The causal fungal pathogen of the disease belongs to the same species as that causing rice blast disease [6]. Disease symptoms first appear as small brown spots on leaves and stems, and develop into water-soaked spots that further progress into round or oval lesions with gray centers and dark-brown margins. If *M. oryzae* heavily infects leaves of susceptible genotypes, the infected leaves die, and small seedlings are killed within a matter of days.

A diversity of resistant phenotypes against the GLS has been observed in ryegrass species, and some resistant genotypes have been found from cultivars and experimental lines in both perennial ryegrass [5,7] and Italian ryegrass [8,9]. In addition, this resistance may be controlled by a few major gene loci [5] with high levels of heritability [5,7], suggesting that a breeding program based on recurrent selection should be effective to improve the resistance to GLS in ryegrasses [5].

In this context, we have identified a locus for a GLS resistance gene, *LmPi1*, on linkage group (LG) 5 of Italian ryegrass [4] and performed targeted mapping of rice expressed sequence tags (ESTs) around the locus using a synteny-based comparative genomics approach [10]. Similarly, Curley et al. [11] reported four quantitative trait loci (QTLs) for GLS resistance on LG 2, 3, 4, and 6 from a mapping population derived from parental clones of Italian × perennial ryegrass hybrids. These achievements are expected to promote breeding programs for GLS-resistant cultivars in ryegrasses, because breeders can easily screen GLS-resistant genotypes using genetic molecular markers linked tightly to the above-mentioned resistance loci.

However, because the breakdown of resistance controlled by a few major genes is a known phenomenon in rice blast disease [3], the durability of the previously identified resistance gene loci in ryegrasses cannot be assured, and other novel loci for GLS resistance should be identified and used for developing durable resistant cultivars against GLS in the future.

Thus, we attempted to identify a novel genetic locus for GLS resistance from an F_1_ population by bulked segregant analysis [12] and a synteny-based comparative genomics approach with rice genome information. A genetic linkage map corresponding to ryegrass LG 3 was constructed by bulked segregant analysis with amplified fragment length polymorphism (AFLP) and simple sequence repeat (SSR) markers. Targeted mapping of rice EST-derived markers further enriched the linkage map. QTL analysis with the linkage map and phenotypic data of the F_1_ population detected a resistance gene locus that explained 61.0–69.5% of the phenotypic variance that was influenced and fluctuated by age of leaves inoculated. The position of the resistance gene locus was confirmed to be distinguishable from previously identified GLS resistance gene locus on ryegrass LG 3 reported by Curley et al. [11]. We designated the resistance gene *LmPi2* as a novel gene for GLS resistance in ryegrasses.

## Results

### Evaluation of GLS resistance in the F_1_ population

We conducted two independent inoculations each for the second-youngest leaves still expanding and the third-youngest fully expanded leaves, which are hereafter referred to as young leaves and expanded leaves, respectively, in the F_1_ population (four inoculations in total). We scored after seven days for each inoculation moment according to the rating scale shown in Table 1.

**Table 1:** **Rating scale for phenotypic assessment of gray leaf spot resistance**

**Phenotype**	**Score**	**Symptoms**
Resistant	0	No visible symptoms
	1	Dark-brown, non-sporulating lesions
	2	Expanding, dark-brown, non-sporulating lesions
Susceptible	3	Small circular or diamond-shaped lesions with sporulating areas
	4	Large expanding lesions with sporulating areas

See details and corresponding photographs in Takahashi et al. [13].

**Figure 1:**
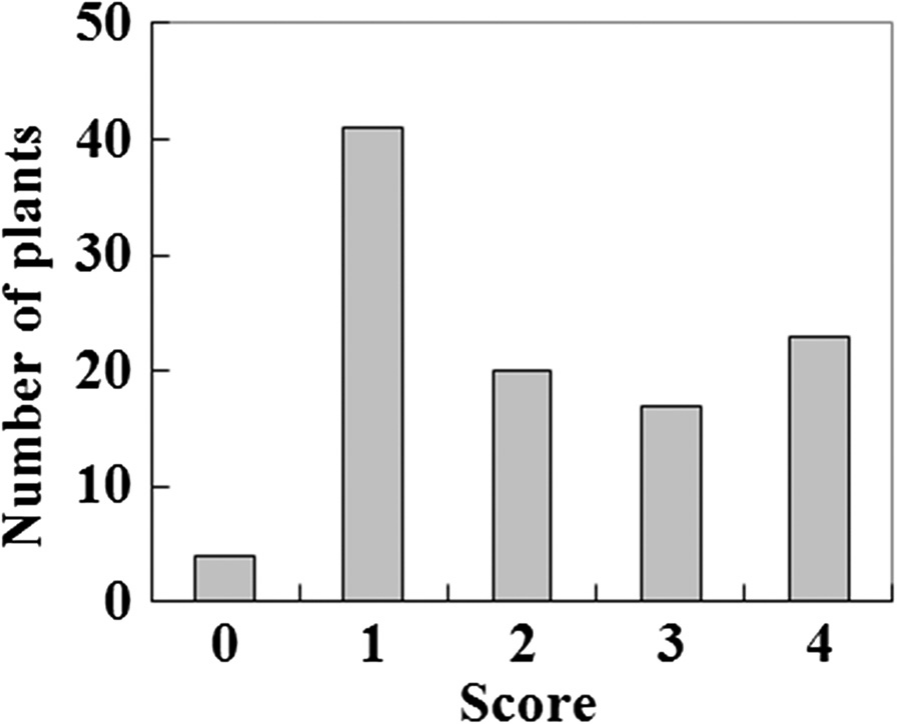
**Frequency distribution of gray leaf spot severity in an Italian ryegrass F**_**1**_ **population derived from cv. ‘Surrey’ (resistant - score 1) and cv. ‘Minamiaoba’ (susceptible - score 4).** Average phenotypic values from four inoculation experiments are shown.

Averaged phenotypic values for each genotype were calculated from each datum of the experiment with young or expanded leaves, and all four experiments. Actual phenotypic segregations in the F_1_ population were 59 resistant (scores 0–2) and 46 susceptible (scores 3–4) plants in the young leaf experiment, 72 resistant and 33 susceptible plants in the expanded leaf experiment, and 65 resistant and 40 susceptible plants as the averages of all four inoculations (Figure 1). The segregation ratios were not different from 1:1 in the young leaf experiment (*χ*^2^ = 1.61, *P* = 0.20) and 3:1 in the expanded leaf experiment (*χ*^2^ = 2.31, *P* = 0.13); however, the segregation ratio for averages of all four inoculations was statistically different from both 1:1 and 3:1. These results indicated that the GLS resistance in the F_1_ population was possibly controlled by one or two dominant genes.

There was a significant correlation (*P* < 0.01) among all GLS severity of the different leaf ages and inoculation moments (Table 2). In particular, higher correlation coefficients were obtained between the results of the same leaf stage (Table 2). Repeated-measures analysis of variance (ANOVA) indicates that there were significant differences (*P* < 0.01) among genotypes in all inoculations for GLS severity; however, the differences were not significant between the inoculations within the same leaf age (Table 3a). Two-way ANOVA using the same data set with that of the above-mentioned repeated-measures ANOVA revealed significant differences (*P* < 0.01) among genotypes and leaf ages for GLS severity, and a significant interaction (*P* < 0.01) between genotype and leaf age (Table 3b). The percentages of broad-sense heritability calculated from the results of the repeated-measures ANOVA shown in Table 3a were 70.1%, 77.9%, and 66.5% for the young leaf experiment, the expanded leaf experiment, and all four inoculations, respectively.

**Table 2:** **Pearson’s correlation coefficients among gray leaf spot assessments in an Italian ryegrass F**_**1**_ **population derived from cv. ‘Surrey’ (resistant) and cv. ‘Minamiaoba’ (susceptible)**

**Experiment** ^**a)**^	**Young leaves 1st**	**Young leaves 2nd**	**Expanded leaves 1st**	**Expanded leaves 2nd**
Young leaves 1st	1			
Young leaves 2nd	0.70	1		
Expanded leaves 1st	0.66	0.58	1	
Expanded leaves 2nd	0.67	0.61	0.78	1

^a)^1st and 2nd indicate first and second inoculation experiment, respectively.

All coefficients were obtained with *P* < 0.01.

**Table 3:** **Repeated-measures ANOVA (a) and two-way ANOVA (b) for gray leaf spot assessments in an Italian ryegrass F**_**1**_ **population derived from cv. ‘Surrey’ (resistant) and cv. ‘Minamiaoba’ (susceptible)**

		**Factor** ^**a)**^	**Sum of squares**	***Df*** ^**b)**^	**Mean square**	***F*** ^**c)**^
(a)	Young leaves^d)^	Genotype	407.50	104	3.92	5.68^*^
		Inoculation	1.22	1	1.22	1.77
		Error	71.78	104	0.69	
		Total	480.50	209		
	Expanded leaves	Genotype	367.70	104	3.54	8.05*
		Inoculation	0.80	1	0.80	1.83
		Error	45.70	104	0.44	
		Total	414.20	209		
	Total	Genotype	668.53	104	6.43	8.95*
		Inoculation	34.62	3	11.54	16.06*
		Error	224.13	312	0.72	
		Total	927.28	419		
(b)	Total	Genotype	668.53	104	6.43	11.30*
		Leaf age	32.59	1	32.59	57.28*
		Genotype × Leaf age	106.66	104	1.03	1.80*
		Error	119.50	210	0.57	
		Total	927.28	419		

^a)^All factors were recognized as fixed effect.

^b)^Number of degrees of freedom.

^c)^Value of F-distribution.

^d)^The data for young leaves have also been shown in Takahashi et al. [13].

*P < 0.01.

### Detection of a GLS resistance gene

To detect the major gene locus, we employed bulked segregant analysis [12] because we succeeded in detecting a major gene, *LmPi1*, for GLS resistance using the method in a previous study [4]. First, we used 64 primer combinations for AFLP and identified two markers, E38/M47 and E32/M59, which showed specific signals in both the resistant parent and the resistant bulk. Preliminary genetic linkage analysis and subsequent QTL analysis indicated that the two markers were linked together and were associated with GLS resistance (data not shown). This result encouraged us to further progress the analysis using SSR markers from a ryegrass reference map developed by Hirata et al. [14] to identify the LG containing the resistance locus. Bulked segregant analyses with 218 SSR markers revealed four markers that showed specific signals in the resistant parent and resistant bulk. Of these, two markers 08-08B and 9-12A have already been reported to locate on ryegrass LG 3 with a relatively close genetic distance between them, whereas the other two markers, 12-01E and 17-01H, have been reported to locate on LG 6 and LG 7, and LG 2, respectively [14]. From these results, we predicted that the resistance gene locus might be in ryegrass LG 3. Nevertheless, all four resistant bulk-specific SSR markers were selected for map construction of LG 3.

The two resistant bulk-specific AFLP and four SSR markers, and 38 other SSR markers that have been reported to locate on ryegrass LG 3 [14], were then used to construct a genetic linkage map corresponding to ryegrass LG 3 with deoxyribonucleic acid (DNA) isolated from individuals from the F_1_ population. Segregation types of the banding patterns for the AFLP and SSR markers are shown in Table 4. As a result, the genotypic data of the F_1_ population obtained from two AFLP and 29 SSR markers were selected for map construction of LG 3.

**Table 4:** **Segregation types for the different markers analysis conducted in an Italian ryegrass F**_**1**_ **population derived from cv. ‘Surrey’ (resistant) and cv. ‘Minamiaoba’ (susceptible)**

**Markers**	**No. of markers analyzed**	**Segregation types** ^**a)**^					**No. of markers omitted** ^**b)**^	**No. of polymorphic markers**
		**lm × ll**	**nn × np**	**ef × eg**	**ab × cd**	**hk × hk**		
AFLP	2	2	0	0	0	0	0	2
SSR	40	19	5	5	0	0	11	29
Rice EST-derived	76	7	9	8	1	2	49	27
Grass anchor probe-derived	7	1	1	0	0	0	5	2

^a)^Parental genotypes were coded in accordance with JoinMap 4 [18].

^b)^Markers that showed unclear, non-segregated, and unexpected banding patterns in the mapping population, or were monomorphic between parents of the mapping population, were omitted.

### Targeted mapping around the locus for GLS resistance

LG 3 of ryegrass species are syntenic to rice chromosome (Chr) 1 [15,16]; therefore, we selected rice EST clones from the rice EST map of Chr 1 [17] at a genetic distance of approximately every 5 centimorgan (cM) or less, as far as possible. Furthermore, grass anchor probes that locate on LG 3 of ryegrass [11] were selected. In total, 76 rice EST clones and seven anchor probes were selected, and primer pairs were designed from these. Among the rice EST clones, 51 primer pairs (67.1%) successfully amplified clear polymerase chain reaction (PCR) products from the female and/or male parent. Thirty-seven primer pairs (48.7%) successfully amplified fragments that were polymorphic in the F_1_ population in single-strand conformation polymorphism (SSCP) analysis. Similarly, two primer pairs (28.6%) derived from grass anchor probes successfully amplified clear PCR products from the female and/or male parent; both were polymorphic between the parents in SSCP analysis. Most of the SSCP analyses showed multiple bands (data not shown). However, most banding patterns from the SSCP analyses could be categorized into the five segregation types shown in Table 4. Detailed results of the SSCP analysis are also shown in Additional file 1. In total, 27 rice EST-derived markers and two grass anchor probe-derived markers, which were categorized into the five segregation types, were used for the genetic map construction of LG 3.

### Construction of a genetic linkage map

**Figure 2:**
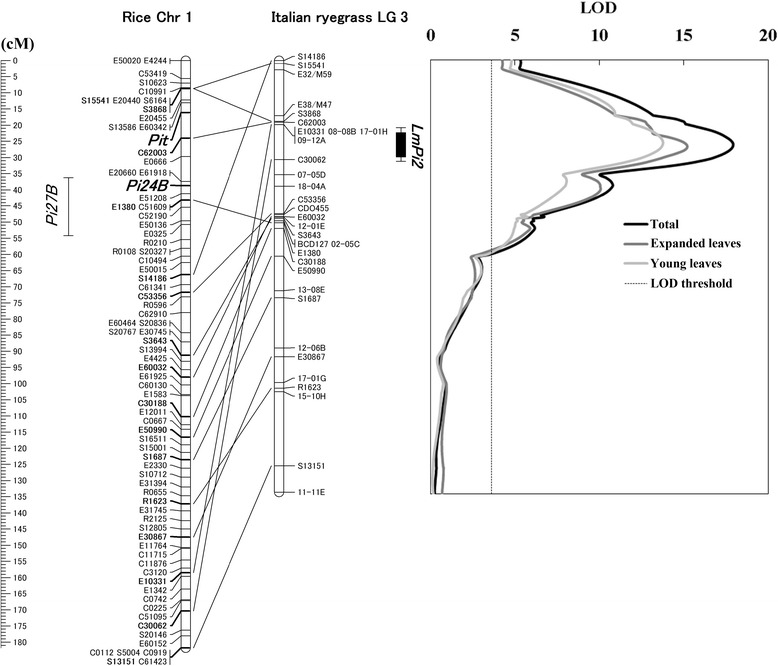
**Comparative rice Chr 1 – Italian ryegrass LG 3 genetic maps and a LOD score plot obtained by QTL analysis.** Markers on the rice Chr 1 genetic map were selected from the rice EST map [17] to develop markers with intron-scanning primers. The developed rice EST-derived markers are indicated by EST clone names (e.g., S14186) provided by the Rice Genome Project (http://rgp.dna.affrc.go.jp/E/Publicdata.html). AFLP markers are indicated by following the nomenclature of AFLP primer enzyme combinations of Key genes (e.g.. E32/M59). SSR markers are indicated by the names (e.g., 08-08B) given by Hirata et al. [14]. Comparative loci between rice and Italian ryegrass are shown in bold on the rice Chr 1 genetic map and are connected by solid lines. Locations of rice disease resistance gene loci on the rice Chr 1 reviewed by Ballini et al. [19] are shown in italics. Genetic distances are measured in centimorgans (cM) against the ruler on the left side of the figure. The linkage map for the ryegrass LG 3 was used for QTL analysis. The graph on the right side of the linkage map shows LOD score plots obtained by interval mapping. The light gray, gray, and black curves represent score plots for young leaves, expanded leaves, and total data obtained from four inoculation experiments, respectively. A broken vertical line indicates a LOD significance threshold level, 3.6, calculated by a permutation test (*P* < 0.05) with 1000 repetitions. The position of *LmPi2* is shown with an inner and outer vertical bar for 1-LOD and 2-LOD support interval, respectively. The position of *LmPi2* and the LOD significance threshold level were calculated based on a result of QTL analysis calculated with the total data obtained from four inoculation experiments.

AFLP, SSR, and SSCP data were analyzed by JoinMap 4 [18]. The analysis yielded a major group with a logarithm of odds (LOD) threshold of 2.0 with 57 markers, and we succeeded in constructing a genetic linkage map covering 133.6 cM with two AFLP-, two grass anchor probe-, 12 SSR-, and 16 rice EST-derived markers (Figure 2).

Of the SSR markers selected by bulked segregant analyses, the two markers 12-01E and 17-01H previously assigned to LG 6 and LG 7, and LG 2, respectively [14], were also integrated into the linkage map; however, the order of other SSR markers in the linkage map was identical to other LG 3 maps from previous studies [14,20], indicating that the linkage map of the present study accurately represents LG 3 of ryegrasses. Significant collinearity (Spearman’s rank correlation rho = 0.64, *P* < 0.01) between the ryegrass LG 3 and rice Chr 1 genetic linkage map [17] was also observed using the information of the order of the rice EST-derived markers (Figure 2).

### Identification of a novel locus for GLS resistance

QTL analyses with the linkage map and phenotypic data of GLS severity revealed a locus for GLS resistance in the LG 3 (Figure 2). Three LOD score plots obtained with the phenotypic data of young leaves, expanded leaves, and total data obtained from four inoculation experiments had similar shapes and showed peaks at almost the same genetic position (Figure 2). The highest LOD scores for young leaves, expanded leaves, and total data obtained from four inoculation experiments were 13.8, 15.2, and 17.9, respectively. Proportions of phenotypic variance explained by the QTL at the highest LOD scores for young leaves, expanded leaves, and total data obtained from four inoculation experiments were 61.0, 68.1, and 69.5%, respectively. Estimated additive effects contributed by the resistant parent at the same genetic positions with those of the highest LOD scores for young leaves, expanded leaves, and total data obtained from four inoculation experiments were −1.09, −0.99, and −1.05, respectively. The position of the GLS resistance gene locus was predicted by 1-LOD and 2-LOD support intervals of the LOD score plot, based on the total data obtained from four inoculation experiments. The rice EST-derived marker C30062 was found to be the closest marker to the resistance locus (Figure 2). Since the proportions of phenotypic variance explained by the QTL were similar to percentages of the above mentioned broad-sense heritability, most of genetic factors for the resistance phenotype against GLS in the F_1_ population were thought to be explained by a function of the detected single gene locus nevertheless the segregation ratios of resistance to susceptibility in the F_1_ population suggested that one or two major loci are involved with the resistance*.* We designated the putative gene for the resistance locus as *LmPi2*, because this is the second major locus for GLS resistance after *LmPi1* [4] in Italian ryegrass.

## Discussion

The severity of GLS is influenced by environmental factors, such as temperature and humidity [21,22]. Accordingly, phenotype evaluations for populations in QTL analysis should be conducted multiple times, and environmental conditions during the phenotype evaluations should be as stable as possible to increase the heritabilities of target traits, because higher heritabilities will lead to more accurate estimations during the analysis. Thus, we employed the filter-paper method [13], by which we could evaluate GLS severity of an F_1_ population four times under fully controlled inoculation conditions *in vitro.* This overcame the high lethality of the GLS and annuality of the Italian ryegrass, because the method does not require whole plants, and only requires detached leaves of young seedlings. A correlation analysis for inoculation experiments showed strong correlations, especially between the results for the same leaf age (Table 2). Repeated-measures ANOVA showed no significant difference between inoculation experiments within the same leaf age (Table 3a), indicating the high repeatability of the filter-paper method.

Frequency distribution of the disease severity of the F_1_ population in the present study was skewed toward resistance in the expanded leaves compared with young leaves. That is, segregation ratios of resistance to susceptibility in young leaves and in expanded leaves were not different from 1:1 and 3:1, respectively. In GLS in ryegrasses, the more severe susceptibility of younger seedlings [23], and mixing of lesion types that tends to be more severe on younger leaves on the same plant [11], have been reported. These reports and the results of the present study suggest that it is important to use the same leaves or leaves at least under the same physiological condition for repeated evaluations of GLS severities in each plant.

The segregation ratios of resistance to susceptibility suggested that one or two major loci are associated with resistance in the F_1_ population. We then used a three-step analysis to detect the resistant gene locus: (1) A genome-wide survey of the target locus by AFLP analysis; (2) identification of the LG containing the target locus using SSR markers; and (3) targeted mapping of the target locus by synteny-based comparative genomics approach with rice EST-derived intron-scanning primers. These processes rapidly detected the resistance gene in the F_1_ population and identified a locus on LG 3 comprising the resistance gene by the bulked segregant AFLP and SSR analysis, respectively. Subsequent synteny-based targeted mapping with rice EST-derived primer pairs effectively produced an enhanced map of LG 3 covering 133.6 cM (Figure 2).

In the targeted mapping, 67.1% of the rice EST-derived primer pairs amplified PCR products from the male and/or female parent. The efficiency was almost the same as our previous study, where 64.3% of intron-scanning primer pairs derived from ESTs on a rice Chr 9 syntenic to a ryegrass LG 5 amplified clear PCR products [10]. Although it is not clear how much sequence similarity there is between the rice EST-derived primers used in this study and target ryegrass genomic sequences, improvement of the primers using a strategy of conserved three-prime end region (COTER) primers, which have perfect similarity to target genomic sequences in eight bases at their 3’ ends and thus can be highly transferable markers among temperate forage grasses [24], might further increase the efficiency.

QTL analysis with phenotypic values for GLS resistance in the F_1_ population succeeded in detecting a major *LmPi2* locus for a GLS resistance on the constructed map of LG 3 (Figure 2). Although the maximum LOD scores for GLS resistance obtained from phenotypic values of young leaves, expanded leaves, and total data obtained from four inoculation experiments were fluctuated by age of leaves inoculated, those were observed at almost the same position on the LG 3 map (Figure 2), suggesting that resistance conferred by the *LmPi2* locus is functional at various leaf ages.

Curley et al. [11] reported high broad-sense heritabilities of GLS resistance against an isolate GG9 and low percentages of total phenotypic variance explained for three QTLs with ranges of 0.895–0.932 and 32.3–53.0%, respectively, in their mapping population. They mentioned that the reason why the percentages of total phenotypic variance explained were lower than those expected from the broad-sense heritabilities might result from additional undetected low-effect QTLs or distorted segregation around regions of the most significant QTLs. By contrast, in this study, percentages of broad-sense heritability and of phenotypic variance explained at the highest LOD score of the *LmPi2* locus, which were calculated with total data obtained from four inoculation experiments, were 66.5% and 69.5%, respectively. Although we only constructed the LG 3 map to detect the *LmPi2* locus, these values are very similar, indicating that most of the genetic factors for the resistance phenotype against GLS in the F_1_ population can be explained by a function of the single *LmPi2* gene.

*LmPi2* locus is clearly distinguishable from a previously identified resistance gene locus for *LmPi1* [4,10], because they each locate on a different LG. Conversely, one of the four QTLs detected by Curley et al. [11] was also reported to locate on the same LG as *LmPi2* locus. Unfortunately, we could not directly distinguish between that QTL and *LmPi2* locus since most grass anchor probe-derived markers, some of which are located around the QTL of Curley et al. [11], could not be added to our map of LG 3 because of unsuccessful PCR amplification in this study (Additional file 1). Thus, substantively, we confirmed the genetic distance between these genetic loci using information from an LG 3 map reported by Hirata et al. [14]. On that map, the closest grass anchor probe, CDO460, linked tightly to the QTL of Curley et al. [11] but is genetically over 25 cM distant from SSR markers, 08-08B and 09-12H, both of which are closely linked to *LmPi2* on our map of LG 3 (Figure 2). This means the *LmPi2* locus is probably different from the QTL detected by Curley et al. [11] and thus we suggest it as a novel locus for GLS resistance.

Plant disease resistance genes and resistance gene analogs (RGAs) often form clusters in genomes [25-28]. Both *LmPi2* locus and the above-mentioned QTL of Curley et al. [11] are on ryegrass LG 3, and one of the isolated ryegrass RGAs [29] may be located on a corresponding region between the QTLs [30]. Similarly, as shown in Figure 2, rice Chr 1 is known to include some genes for rice blast resistance around a syntenic region to the ryegrass LG 3. From these, a homeologous cluster for disease resistance might be formed around the syntenic region in both the ryegrass and rice, although disruption of synteny between cereal grasses is often revealed in resistance gene loci [31-33].

Most genetic factors for resistance in the F_1_ population used in this study could be explained by *LmPi2* locus; therefore, the resistance locus will be useful to develop GLS-resistant cultivars in combination with *LmPi1* locus [4] and the QTLs detected by Curley et al. [11]. One major concern has, however, been revealed by the breeding histories of blast-resistant cultivars in rice: the breakdown of resistance controlled by a few major genes is one of the most important issues in the development of blast-resistant cultivars in rice [3]. Both GLS in ryegrasses and rice blast disease are caused by a common pathogenic species, *M. oryzae* [6]; therefore, it is reasonable to predict that the same phenomenon might occur in GLS-resistant cultivars if their resistance were controlled by a few resistance genes. Development of convertible multiple line cultivars composed of exchangeable multiple isogenic lines, each containing one major resistance gene, might be one way to develop durable resistant cultivars against GLS, as has been the case for rice [3], although the breeding systems for ryegrasses are quite different from those of rice because of the nature of outcrossing.

## Conclusions

We identified a genetic locus for GLS resistance from a single cross-derived F_1_ population of Italian ryegrass (*L. multiflorum* Lam.) by bulked segregant analysis. The resistance locus was detected on ryegrass LG 3 of ryegrasses and explained 61.0–69.5% of the phenotypic variance that was influenced and fluctuated by age of leaves inoculated. Since the phenotypic variance and percentages of broad-sense heritability were similar, most of the genetic factors for the resistance phenotype against GLS in the F_1_ population can be explained by a function of the single resistance locus. The resistance locus was confirmed as a novel GLS resistance locus, because the genetic position of the locus was different from other known loci for GLS resistance. We designated the putative gene for the novel resistance locus as *LmPi2*.

## Methods

### Plant materials

An F_1_ population of Italian ryegrass (*L. multiflorum* Lam.) was generated from a single cross between two heterozygous individuals: a GLS-resistant individual of cv. ‘Surrey’ as the female parent and a GLS-susceptible individual of cv. ‘Minamiaoba’ as the male parent. The cv. ‘Surrey’ and cv. ‘Minamiaoba’ are registered as PI 593651 in the Germplasm Resources Information Network (GRIN; http://www.ars-grin.gov/) and as JP 67746 in the National Institute of Agrobiological Sciences GeneBank (NIAS GeneBank; https://www.gene.affrc.go.jp/index_en.php), respectively.

The F_1_ population, comprising 105 individuals, had been used previously to establish the filter-paper method for evaluation of GLS resistance in Italian ryegrass [13]. Seeds were sown in soil in 96-well trays (8 × 12 wells; 28 × 40 cm), and grown in a glasshouse at 25°C. Total genomic DNAs of the F_1_ population were extracted from leaves with a DNeasy plant mini kit (Qiagen, Hilden, Germany) and were subjected to polymorphism analyses, as mentioned below.

### Experimental design

For evaluating GLS resistance, we employed a repeated measures design with four inoculations composed of two independent inoculations each with the second-youngest leaves still expanding and the third-youngest fully expanded leaves in the F_1_ population. That is, we detached two each of the second-youngest and the third-youngest leaves from each genotype, and subjected the four detached leaves to independent inoculations to make in total four inoculations composed of two times each for the second-youngest and the third-youngest leaves per genotype. This experimental design with the associated samples allowed us to test the significance of the factors genotype and inoculation. In addition, since the leaves of each genotype were separately placed in different culture dish and subjected to each experiment in randomized inoculation order, we also tested the significance of the factor leaf age and interaction between genotype and leaf age.

### Preparation of conidial suspensions

A single-postule isolate of *M. oryzae* obtained from a natural infection of Italian ryegrass in Yamaguchi Prefecture, Japan [4] was used. The isolate was grown on culture medium containing 5% (w v^−1^) oatmeal, 2% (w v^−1^) sucrose, and 3.5% (w v^−1^) agar and incubated in the dark at 25°C for 10 days. Aerial mycelia were scraped off the surface with a brush. Conidiation was induced by exposing the mycelia to near-ultraviolet light at 25°C for 5 days, and the conidia were suspended in distilled water. The final density of conidia and the final concentration of the surfactant Tween 20 in the inoculum were adjusted to 5 × 10^4^ conidia mL^−1^ and 0.01% (v v^−1^), respectively.

### Artificial inoculation

We used the filter-paper method [13] to evaluate GLS resistance in the F_1_ population. That is, leaf segments 2.5 cm long were detached from seedlings at the two- or three-tiller stage, and were placed, abaxial side up, in Petri dishes containing 0.7% (w v^−1^) agar supplemented with 40 mg L^−1^ benzimidazole. Ten microliters of conidial suspension was dropped onto a 2 × 15 mm rectangle of filter paper (No. 5B; Toyo roshi kaisha, Tokyo, Japan). The inoculated surface of the filter paper was then placed in contact with the leaf. The Petri dishes were sealed with Parafilm (PM-996; Bemis Company, Neenah, WI, USA) and incubated for 24 h in the dark at 25°C. The filter paper was then removed, and the Petri dish was sealed again with Micropore surgical tape (1530-0; 3 M Health Care, Saint Paul, MN, USA). The inoculated leaves were further incubated for 7 days under short-day conditions (8 h light/16 h dark) at 25°C; light with a photon flux intensity of 100 μmol m^−2^ s^−1^ at plant level was provided by fluorescent lamps (FL40SEX-N-HG; NEC lighting, Tokyo, Japan). After the incubation, disease symptoms were evaluated according to the rating scale shown in Table 1.

### Bulked segregant analysis

Ten resistant (scores range 0–1) and 10 susceptible (score 4) individuals of the F_1_ population were selected with an average score obtained with four independent evaluations of the GLS resistance. Genomic DNAs from these resistant and susceptible individuals were then mixed in equal proportions to construct resistant and susceptible bulks, respectively, and subjected to AFLP and SSR analyses.

### AFLP analysis

AFLP analyses were carried out with the IRDye fluorescent AFLP kit for large plant genome analysis (LI-COR, Lincoln, NE, USA). We analyzed 64 AFLP selective primer combinations: *Eco*RI + AX_1_X_2_/*Mse*I + CX_3_X_2_ (X_1_ = A or C; *X*_2_ = A, C, G or T; X_3_ = A or T). The PCR products were separated by electrophoresis through 6% (w v^−1^) denaturing acrylamide gels in a LI-COR DNA analyzer (LI-COR), according to the manufacturer’s instructions.

### SSR analysis

We conducted SSR analysis with 218 primer combinations that were assigned to locations on the seven LGs corresponding to the haploid Italian ryegrass karyotype [14]. PCR was performed in a GeneAmp PCR system 9700 (Applied Biosystems, Foster City, CA, USA) with a 10-μL reaction mixture containing 0.05 μL Hot Star Taq (5 units μL^−1^; Qiagen), 1 μL 10× PCR buffer, 0.4 μL 25 mM MgCl_2_, 0.8 μL dNTPs (2.5 mM each), 0.2 μL each primer (20 pmol μL^−1^), 20 ng genomic DNA and 5.35 μL sterile distilled water. After the first treatment of the reaction mixture at 95°C for 15 min, the following PCR programs were performed: 10 cycles of 94°C for 15 s, 65–56°C (−1°C per cycle) for 15 s, and 72°C for 2.5 min; 30 cycles of 94°C for 15 s, 55°C 15 s, and 72°C for 1 min; 72°C for 7 min. The PCR products were electrophoresed through precast polyacrylamide gel (GeneGel Excel 12.5/24; GE Healthcare, Buckinghamshire, UK) in a Peltier temperature-regulated electrophoresis unit (GenePhor; GE Healthcare) with an electrophoresis power supply (EPS3501XL; GE Healthcare), in accordance with the manufacturer’s instructions. Sample buffer was made as follows: 23 mL distilled water, 250 μL 0.1 M EDTA, and 500 μL 0.5 M Tris were mixed and adjusted to pH 7.5 using acetic acid, and then 1.25 mL 1% (w v^−1^) xylene cyanol and 10 mg bromophenol blue were added. Two microliters of the sample buffer were mixed with 4 μL of PCR product. The mixture was loaded onto a polyacrylamide gel, which was temperature regulated at 25°C, and electrophoresed for 80 min at 600 V, 25 mA, and 15 W. Silver staining was used to visualize the isolated PCR products, using a silver staining kit (GE Healthcare) in a Hoefer automated gel stainer (GE Healthcare).

### Design of intron-scanning primers

Synteny-based genetic mapping was used for marker saturation around a target resistance gene locus by a procedure previously mentioned by Takahashi et al. [10]. Synteny between ryegrass and rice has been demonstrated by other research groups [15,16]; therefore, we selected rice EST clones from a Chr that is syntenic to a target LG of ryegrass from public EST map data [17] in the online database of the Rice Genome Research Program (RGP; http://rgp.dna.affrc.go.jp/E/). Subsequently, genomic clones [P1-derived artificial Chr (PAC) clones] that contained the nucleotide sequence information for the selected EST clones were retrieved from the Rice Annotation Project Database (RAP-DB; http://rapdb.dna.affrc.go.jp/) [34]. A coding sequence (CDS) of the EST clone was concomitantly obtained with the nucleotide sequence features of the retrieved PAC clone. The exon/intron structure of the target gene was predicted by generating CDS-to-PAC clone sequence alignments with Spidey [35], an online tool for mRNA-to-genome alignment (http://www.ncbi.nlm.nih.gov/IEB/Research/Ostell/Spidey/). Primer pairs in the predicted exon regions were designed to amplify across predicted intron regions using the primer analysis software, OLIGO v. 6.7 (Molecular Biology Insights, Cascade, Chico, CA, USA). PCR was performed in a GeneAmp PCR system 9700 (Applied Biosystems) with a 10-μL reaction mixture containing the same components as those in SSR analysis. After the first treatment of the reaction mixture at 95°C for 15 min, the following PCR programs were performed: two cycles of 94°C for 1 min and 72°C for 2.5 min; two cycles of 94°C for 1 min and 68°C for 2.5 min; two cycles of 94°C for 1 min, 65°C for 30 s, and 72°C for 2 min; and 30 cycles of 94°C 1 min, 55°C for 30 s, and 72°C for 2 min. The PCR products were then subjected to SSCP analysis (see below).

### Design of primers from grass anchor probes

PCR primers were also designed from the grass anchor probes developed by Van Deynze et al. [36]. That is, sequence data of each probe were retrieved from GenBank (http://www.ncbi.nlm.nih.gov/genbank/). Primer pairs were designed from the obtained sequences using OLIGO v. 6.7 (Molecular Biology Insights). PCR was performed using the same procedure as that for the above-mentioned intron-scanning primers. The PCR products were subjected to the SSCP analysis, as described below.

### SSCP analysis

SSCP analysis was carried out with the same precast polyacrylamide gel and apparatus used for SSR analysis. The denaturing solution was made in a ca. 25-mL total volume containing 23.75 mL 99% formamide, 1.25 mL 1% (w v^−1^) xylene cyanol, and 10 mg bromophenol blue. To denature the PCR products, equal amounts of PCR products and denaturing solution were mixed to make 6 μL of mixture. The mixture was treated at 95°C for 5 min to denature the DNA and was then cooled rapidly on ice. The denatured sample was loaded onto a polyacrylamide gel, which was temperature-regulated at 5 or 15°C, and electrophoresed for 100 min at 600 V, 25 mA, and 15 W. Silver staining visualized the isolated PCR products, as mentioned in the SSR analysis.

### Construction of a genetic linkage map

Polymorphic markers were scored in each individual of the F_1_ population. The following segregation types were adopted: locus heterozygous in either female or male parent representing two alleles (lm × ll or nn × np), locus heterozygous in both parents representing two alleles (hk × hk), and locus heterozygous in both parents representing three (ef × eg) or four alleles (ab × cd), where the parental genotypes were coded according to JoinMap 4 [18]. The segregation types that were heterozygous in both parents were used as bridge markers. For map construction of LG 3, the segregation data were input and calculated with the algorithm for cross pollination (CP) population type codes in JoinMap 4, and genetic distances were calculated by Haldane’s mapping function. All other calculation conditions of JoinMap 4 were used at default settings. The genetic linkage map was drawn with MapChart 2.2 software [37].

### QTL analysis

The putative location of a resistance gene on the genetic linkage map obtained with the CP population type codes in JoinMap 4 was determined with both genotypic and phenotypic data of the F_1_ population by simple interval mapping in MapQTL 5 [38]. A LOD threshold to declare a significant QTL was also determined by a permutation test (*P* < 0.05) with 1000 replications, in the software. Genetic effects of the detected QTL were also estimated by conducting two-way pseudo-testcross analysis [39] where marker data was separated into two meioses and converted to doubled haploid population codes as described by Van Ooijen [40].

### Statistical analysis

Pearson’s correlation coefficient and chi-squared goodness-of-fit tests were calculated to analyze the phenotypic data of the F_1_ population. Repeated-measures ANOVA and two-way ANOVA, and Spearman’s rank correlation coefficient were also calculated to analyze the phenotypic data of the F_1_ population and the collinearity of genetic maps between ryegrass and rice, respectively. All these analyses were conducted in R v. 2.15.2 software [41].

Broad-sense heritability as a ratio between estimated genotypic variance (*σ*^*2*^_*g*_) and phenotypic variance (*σ*^*2*^_*ph*_) was calculated using the formula *h*^*2*^ = *σ*^*2*^_*g*_/(*σ*^*2*^_*g*_ + *σ*^*2*^_*e*_) where the *σ*^*2*^_*e*_ and *σ*^*2*^_*ph*_ are error variance and a total of *σ*^*2*^_*g*_ + *σ*^*2*^_*e*_, respectively*.* The *σ*^*2*^_*g*_ can be obtained as (MS_g_ − *σ*^*2*^_*e*_)/*r* where the MS_g_ is expected mean square of the factor genotype, which is expressed as *rσ*^*2*^_*g*_ + *σ*^*2*^_*e*_, and the *r* is the number of inoculations per genotype.

### Availability of supporting data

The data supporting the results of this article are included as Additional file 1.

## Additional file

Additional file 1:
**Summary of EST clones selected from rice chromosome 1 and grass anchor probes, and results of the SSCP analysis.**
